# Prognostic effect of triglyceride glucose-related parameters on all-cause and cardiovascular mortality in the United States adults with metabolic dysfunction-associated steatotic liver disease

**DOI:** 10.1186/s12933-024-02287-y

**Published:** 2024-06-01

**Authors:** Yu Min, Xiaoyuan Wei, Zhigong Wei, Ge Song, Xin Zhao, Yi Lei

**Affiliations:** 1 Department of Biotherapy, West China Hospital, Sichuan University, Chengdu, 610041 People’s Republic of China; 2 Department of Head and Neck Oncology, West China Hospital, Sichuan University, Chengdu, 610041 People’s Republic of China; 3 Department of Urology, The Affiliated Hospital of Southwest Medical University, Luzhou, 646000 Sichuan People’s Republic of China; 4 Department of Endocrinology and Metabolism, The Affiliated Hospital of Southwest Medical University, Luzhou, 646000 Sichuan People’s Republic of China; 5 Sichuan Clinical Research Center for Nephropathy, Luzhou, 646000 Sichuan People’s Republic of China; 6 Metabolic Vascular Disease Key Laboratory of Sichuan Province, Luzhou, 646000 Sichuan People’s Republic of China

**Keywords:** MASLD, Insulin resistance, TyG index, TyG-WC index, TyG-WHtR index, All-cause mortality, Cardiovascular mortality

## Abstract

**Backgrounds:**

Insulin resistance (IR) plays a vital role in the pathogenesis of the metabolic dysfunction-associated steatotic liver disease (MASLD). However, it remains unclear whether triglyceride–glucose (TyG) related parameters, which serve as useful biomarkers to assess IR, have prognostic effects on mortality outcomes of MASLD.

**Methods:**

Participants in the National Health and Nutrition Examination Survey (NHANES) database from 1999 to 2018 years were included. TyG and its related parameters [TyG-waist circumference (TyG-WC) and TyG-waist to height ratio (TyG-WHtR)] were calculated. Kaplan–Meier curves, Cox regression analysis, and restricted cubic splines (RCS) were conducted to evaluate the association between TyG-related indices with the all-cause and cardiovascular mortality of adults with MASLD. The concordance index (C-index) was used to evaluate the prediction accuracy of TyG-related indices.

**Results:**

A total of 8208 adults (4209 men and 3999 women, median age 49.00 years) with MASLD were included in this study. Multivariate-adjusted Cox regression analysis revealed that high quartile levels of TyG-related indices were significantly associated with the all-cause mortality of participants with MASLD [_TyG_adjusted hazard ratio (aHR) = 1.25, 95% confidence interval (CI) 1.05–1.50, *P* = 0.014; _TyG-WC_aHR for all-cause mortality = 1.28, 95% CI 1.07–1.52, *P* = 0.006; _TyG-WHtR_aHR for all-cause mortality = 1.50, 95% CI 1.25–1.80, *P* < 0.001; _TyG-WC_aHR for cardiovascular mortality = 1.81, 95% CI 1.28–2.55, *P* = 0.001; _TyG-WHtR_aHR for cardiovascular mortality = 2.22, 95% CI 1.55–3.17, *P* < 0.001]. The C-index of TyG-related indices for predicting all-cause mortality was 0.563 for the TyG index, 0.579 for the TyG-WC index, and 0.585 for the TyG-WHtR index, respectively. Regarding cardiovascular mortality, the C-index was 0.561 for the TyG index, 0.607 for the TyG-WC index, and 0.615 for the TyG-WHtR index, respectively. Nonlinear trends were observed between TyG and TyG-WC indices with all-cause mortality of MASLD (*P* < 0.001 and = 0.012, respectively). A non-linear relationship was observed between the TyG index and cardiovascular mortality of MASLD (*P* = 0.025). Subgroup analysis suggested that adults aged < 65 years old and those without comorbidities were more sensitive to the mortality prediction of TyG-related indices.

**Conclusion:**

Findings of this study highlight the predictive value of TyG-related indices, especially the TyG-WHtR index, in the mortality outcomes of adults with MASLD. TyG-related indices would be surrogate biomarkers for the clinical management of MASLD.

**Supplementary Information:**

The online version contains supplementary material available at 10.1186/s12933-024-02287-y.

## Introduction

Nonalcoholic fatty liver disease (NAFLD) is one of the most common chronic liver diseases worldwide, and now affects approximately 35% of the global adult population, which is a 50% increase since the 1990s [1, 2]. The NAFLD causes a series of hepatic and extrahepatic complications, including but not limited to risks for cardiovascular disease (CVD), type 2 diabetes mellitus (T2DM), chronic kidney disease (CKD), and premature death [3, 4]. Additionally, the NAFLD shows a significant negative impact on health-related quality of life (HRQoL) and is responsible for a tremendous economic burden in the US adult population [5, 6]. Most recently, following the Delphi consensus process, the term steatotic liver disease (SLD) was introduced to replace fatty liver disease (FLD), while the term metabolic dysfunction-associated steatotic liver disease (MASLD) emerged as the successor to the term NAFLD [7]. With a broader spectrum of SLD etiologies and an in-depth focus on metabolic dysregulation, a diagnosis of MASLD is more likely to support its epidemiologic impact, biomarker identification, and drug development as well as health policy projects [7].

Cardiometabolic-related factors are observed to play a pivotal role in the occurrence of SLD. In particular, insulin resistance (IR) has been determined to be the central mechanism of the development of MASLD [8, 9]. Chronic IR with hyperinsulinemia could stimulate triglyceride synthesis and accumulation in the liver [10]. Alternatively, the accumulation of triglyceride and free fatty acids in hepatocytes will further promote IR through inflammation, oxidative stress, endoplasmic reticulum stress, and lipotoxicity [11]. The bidirectional relationships between IR and MASLD indicated the need to explore the effects of IR-related indicators on the occurrence and prognosis of MASLD, which would contribute to refining the clinical management of this population [8, 10, 11].

Although the insulin-glucose clamp and intravenous glucose tolerance tests were the gold standard for IR detection [12], the nature of complexity and cost limit their clinical utility [13]. Currently, the homeostasis model assessment of IR (HOMA-IR), constructed from fasting blood glucose (FBG) and fasting insulin levels, has been widely utilized for assessing IR in clinical practice [14]. However, the prediction accuracy of this model might be compromised in patients undergoing insulin treatment or those with non-functioning beta cells [13]. Meanwhile, HOMA-IR presented less sensitivity than other IR indicators to determine the progress of individuals with metabolic-related diseases [15, 16]. It’s noteworthy that the triglyceride–glucose (TyG) index has been determined as a surrogate biomarker reflecting the IR but with a different pathway compared to HOMA-IR [17, 18]. The TyG index showed a stronger correlation with lipid metabolism than HOMA-IR and exhibited superior predictive value for the onset of metabolic-related diseases [18–20]. In particular, emerging evidence demonstrated the prediction value of the TyG index in the development of NAFLD as well as MASLD in diverse populations [17, 21, 22]. Notably, a meta-analysis systemically summarized the predictive value of the TyG index for the diagnosis of metabolic-associated fatty liver disease (MAFLD) [23]. However, evidence on the prognostic effect of the TyG index in adults with MASLD remains sparse [23]. Alternatively, novel indices derived from the integration of the TyG index with adiposity-related indicators, especially in terms of TyG-waist circumference (TyG-WC) and TyG-waist-to-height ratio (TyG-WHtR), have been proposed [25–27]. These new indices are considered to potentially offer a more precise predictive value for disease outcomes compared to the original TyG index [24–26]. However, it remains unclear whether the TyG-WC and TyG-WHtR are correlated with the prognosis of MASLD and present an improved predictive accuracy for the survival of this population.

To fill the research gaps, the goal of the present study is to investigate the associations between TyG, TyG-WC, and TyG-WHtR indices with all-cause and cardiovascular mortality of adults with MASLD, based on a large-scale population-based cohort. This is a preliminary study to explore the links between TyG-related indices and the clinical outcomes of adults with MASLD, which may bring additional benefits to the clinical management of adults with MASLD.

## Materials and methods

### Data source

Data for this study were derived from the National Health and Nutrition Examination Survey (NHANES) database between 1999 and 2018. The NHANES database systematically gathered nationally representative health-related data on the repetitive noninstitutionalized US population, utilizing a stratified, multistage probability sampling design [27]. Specific descriptions of the NHANES database can be found on the website (https://www.cdc.gov/nchs/nhanes/).

### Participants selection

**Fig. 1:**
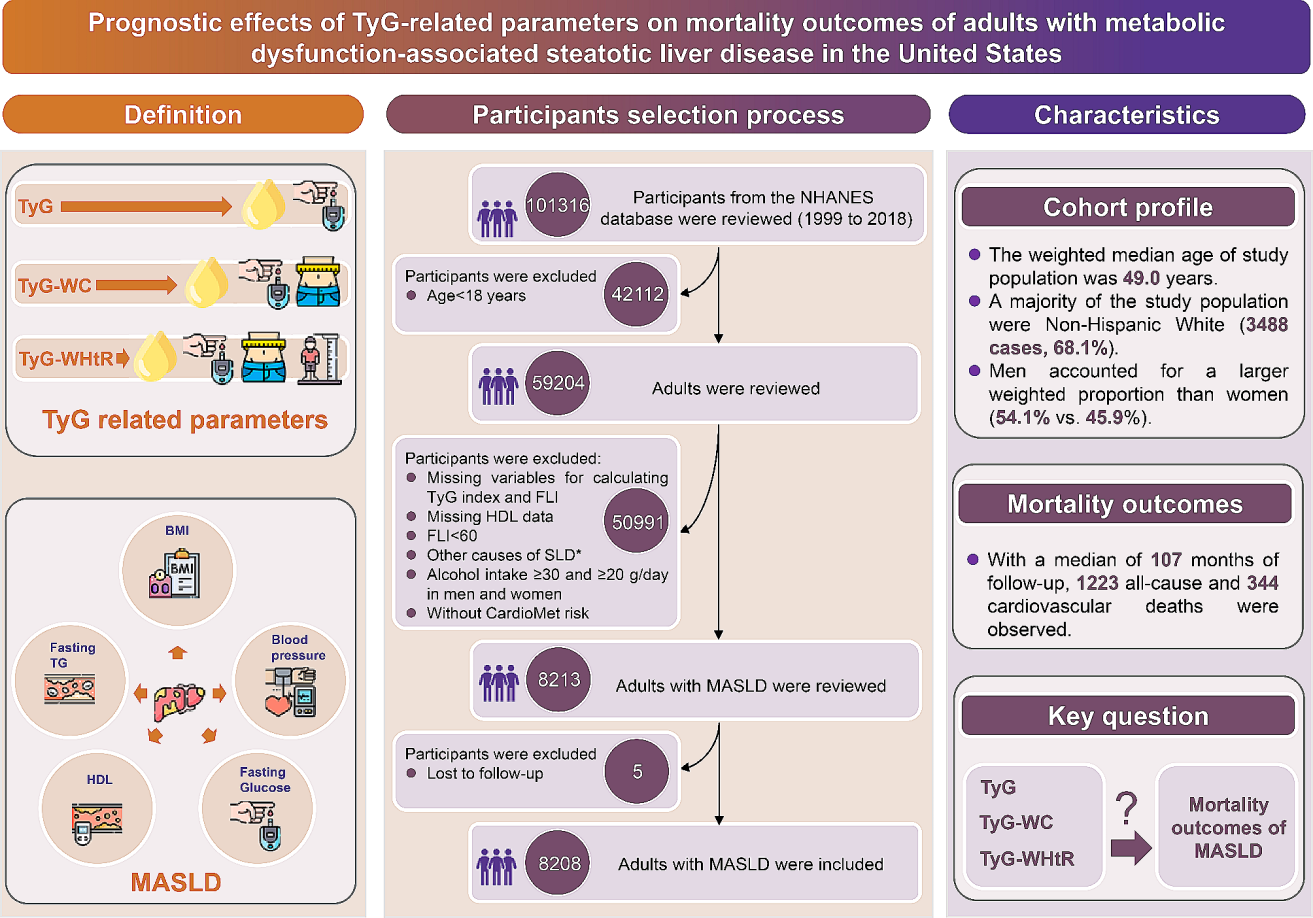
Scheme of the aim of the study and participants selection process. We aim to evaluate the association between varied TyG-related indices with the mortality outcomes of adults with MASLD. MASLD, metabolic dysfunction-associated steatotic liver disease; TyG, triglyceride-glucose; WC, waist circumference; WHtR, waist to height ratio; BMI, body mass index; HDL, high-density lipoprotein; TG, triglyceride. *Other potential causes of SLD: viral hepatitis, autoimmune liver disease, genetic liver diseases, drug- or medication-induced liver disease, and alcohol-related liver disease

To evaluate the correlations between TyG-related indices with all-cause and cardiovascular mortality of adults with MASLD, only participants with the diagnosis of MASLD were included. Therefore, during the ten cycles of interviews (1999 to 2018), 101,316 participants were reviewed. After excluding the participants aged below 18 years old, there were 59,204 participants left. Additionally, participants with missing data on triglyceride (TG) or FBG, body measurements, and metabolic dysfunction-associated factors were excluded. Then, participants presenting clinical features of FLI < 60, with other causes of SLD, a history of moderate to heavy alcohol intake, lacking cardiometabolic risk factors, or lost to follow-up were further excluded. Finally, there were 8208 participants with MASLD were included in this study (Fig. 1).

#### Assessment of MASLD

Direct ultrasonographic assessments of hepatic steatosis were missing in most of the interview cycles. Thus, hepatic steatosis was determined by using the fatty liver index (FLI), which was a reliable tool to evaluate steatotic liver disease (SLD) [28, 29], with high sensitivity and specificity. The equation is listed below [30]:

$$ \begin{aligned} & {\text{FLI}} \\ & \quad = \left( {e^{{0.953 \times {\text{Ln}}({\text{TG}}) + 0.139 \times {\text{BMI}} + 0.718 \times {\text{Ln}}\left( {{\text{GGT}}} \right) + 0.053 \times {\text{WC}} - 15.745}} } \right)/\left( 1 \right. \\ & \quad \quad \left. { + e^{{0.953 \times {\text{Ln}}\left( {{\text{TG}}} \right) + 0.139 \times {\text{BMI}} + 0.718 \times {\text{Ln}}\left( {{\text{GGT}}} \right) + 0.053 \times {\text{WC}} - 15.745}} } \right) \times 100 \\ \end{aligned} $$

TG refers to triglyceride, GGT refers to gamma-glutamyl transferase, BMI refers to body mass index, and WC refers to waist circumference [31, 32]. According to the previous studies, participants with FLI < 60 were considered to have a low probability of hepatic steatosis, while those with FLI ≥ 60 were considered to have a high probability of hepatic steatosis [28, 30]. Therefore, participants with FLI ≥ 60 were diagnosed with SLD. To meet the diagnosis criteria for MASLD according to the Delphi process, participants with viral hepatitis, autoimmune liver disease, genetic liver diseases, drug- or medication-induced liver disease, alcohol-related liver disease, or alcohol intake of ≥ 30 g/day for men and ≥ 20 g/day for women (the daily alcohol intake was obtained from the 24 h dietary recall by the USDA’s automated multiple-pass method [33]) were excluded. Consequently, MASLD was defined as SLD with a combination of the presence of at least one cardiometabolic risk factor:

1. BMI ≥ 25 kg/m^2^ or WC ≥ 94 cm for males and ≥ 80 cm for females;
2. FBG ≥ 100 mg/dL or 2-h post-load glucose levels ≥ 140 mg/dL or hemoglobin A1c ≥ 5.7% or diabetes mellitus (DM) or undergoing hypoglycemic therapy for DM;
3. Blood pressure ≥ 130/85 mmHg or undergoing antihypertensive drug treatment;
4. Fasting plasma triglycerides ≥ 150 mg/dL or undergoing lipid-lowering treatment;
5. Plasma HDL-cholesterol < 40 mg/dL for males and < 50 mg/dL for females or undergoing lipid-lowering treatment [34].

#### TyG-related indices measurements

There were three TyG-related indices measured in the present study. The TyG index was measured by the peripheral blood test [TG, and fasting blood glucose (FBG)] of the participants according to the previous literature [35, 36]. Two variables, including WC and WHtR, were selected as the central obesity indicators in the metabolic dysfunction population [37]. Specifically, the indices of TyG, TyG-WC, and TyG-WHtR were calculated based on the following equations:

1. TyG = ln [TG (mg/dL) × FBG (mg/dL)/2] [38].
2. TyG-WC = ln [TG (mg/dL) × FBG (mg/dL)/2] × waist circumference (cm) [39].
3. TyG-WHtR = ln [TG (mg/dL) × FBG (mg/dL)/2] × waist circumference (cm)/height (cm) [40].

The participants with MASLD were classified into four groups by the quartiles of the TyG index, TyG-WC index, and TyG-WHtR index, respectively, and the group at the 1st quartile was set as the reference.

#### Clinical characteristics and covariates

We collected the demographic characteristics of participants with MASLD from the NHANES database. Specifically, the socioeconomic characteristics including gender (male or female), age, race (Hispanic, non-Hispanic White, non-Hispanic Black, or other races), marital status (not married, married or living with partner), educational level (≤ high school, college, or > college), and poverty income ratio (PIR, < 1.3, 1.3–3.5, or > 3.5) were collected. Besides, the living habits and history of comorbidities including smoking status (never, ever, or current), alcohol use (never, ever, or current), history of DM, cancer, chronic kidney disease (CKD), and cardiovascular disease (CVD) (yes or no) were further collected. In addition, physical and laboratory examinations including WC, height, BMI, energy intake (average kilocalorie derived from two 24-h dietary recall interviews), TG, FBG, total cholesterol (TC), glutamic-pyruvic transaminase (ALT), and aspartate transaminase (AST) were also collected.

### Outcome measurements

The main outcome of this study was the all-cause mortality of the participants with MASLD. The secondary outcome was the cardiovascular mortality of the participants with MASLD. The mortality data for the follow-up population were obtained from the NHANES public-use linked mortality file as of December 31, 2019, which was correlated with the National Center for Health Statistics (NCHS) with the National Death Index (NDI) through a probability matching algorithm. The ICD-10 (International Statistical Classification of Diseases, 10th revision) was used to check the causes of mortality. The period of follow-up was calculated from the date when the interview was initially taken to either the date of the patient’s death or December 31, 2019 [41].

### Statistical analysis

According to the analytic guideline of the NHANES database (https://wwwn.cdc.gov/nchs/nhanes/tutorials/weighting.aspx) (accessed on 4 Mar 2024) for weights in making estimates that results were representative of the U.S. civilian non-institutionalized population. All analyses in this study incorporated sample weights, clustering, and stratification to estimate appropriate variance and ensure national representation of the U.S. population with MASLD.

The Kolmogorov–Smirnov test was used to check the normality assumption distribution of each variable. As shown in Fig. S1, all of the continuous variables were non-normally distributed. Therefore, the continuous variables were presented as median (interquartile range). The categorical variables were presented as numbers (weighted percentage, %) (Fig. S2). Continuous variables between survivors and non-survivors were compared with the Kruskal–Wallis test, whereas categorical variables were compared by using the Chi-Squared test.

Cox proportional hazard models were used to estimate the association of the TyG-related indices with all-cause and cardiovascular mortality of the participants with MASLD. The selections of controlled covariates in the present study were based on previous literature evaluating the survival of MASLD [42–44]. Specifically, model 1 served as the unadjusted analysis. Besides, brief adjustments for age, gender, and race were made in Model 2. In the fully adjusted model, we accounted for age, gender, race, marital status, educational level, poverty income ratio (PIR), smoking status, alcohol use, cancer, CKD, CVD, energy intake, serum levels of TC, ALT, and AST. The concordance index (C-index) [45] was employed to assess the predictive accuracy of both univariate and multivariate-adjusted TyG-related indices for mortality outcomes among participants with MASLD. Kaplan–Meier (KM) curves were used to show the censored data and different survival patterns among the participants with MASLD at different quartiles of the TyG index. Missing data was imputed with multiple imputation methods. To evaluate the dose-effect relationships between TyG-related indices with all-cause and cardiovascular mortality of participants with MASLD, restricted cubic splines (RCS) transformations were applied. The selection of knots for the RCS curves was guided by the minimization of Akaike’s Information Criterion (AIC).

To check the robust associations between TyG-related indices with all-cause and cardiovascular mortality of the participants with MASLD, three sets of sensitive analyses were conducted to validate the main findings. First, we excluded participants who died within 2 years after the interview, which could reduce the potential reverse causality between exposure and outcome. Second, we tested the association between TyG-related indices with mortality outcomes of adults who were interviewed between 1999 and 2006 years, which could check the impact of the different cycles we have chosen on the association. Third, we also checked the mortality prediction value of TyG-related indices in a more generalizable population by applying FLI ≥ 30 as the diagnostic criteria for SLD.

All statistical analyses of the present study were conducted by using the R software (version 4.2.3, https://www.r-project.org/).

## Results

### The baseline characteristics of adults with MASLD

From 1999 to 2018 years, a total of 8208 participants were included in this study. The median age of the study population was 49.00 years and the median BMI was 33.18 kg/m^2^. Male participants (4209 cases, 54.1%) accounted for a relatively higher proportion of the whole study population than females (3999 cases, 45.9%). A majority of the adults with MASLD were non-Hispanic white (3488 cases, 68.1%) and the non-Hispanic black race only accounted for 11.2% of the population (1720 cases). Over 50% of the participants had an educational level above high school. A total of 18.2% of the participants were currently smoking, and 63.0% were currently drinking. Regarding the comorbidities, 17.9% of the participants had a history of DM, 9.3% had CVD, 2.9% had CKD, and 9.8% had cancer. The median TyG index was 8.96, TyG-WC index was 988.27, and TyG-WHtR was 5.86, respectively. With a median of 107 months of follow-up, 1223 all-cause and 344 cardiovascular-related deaths were observed. Non-survivors presented characteristics of male gender, older age, non-Hispanic white race, lower educational levels, unmarried status, concurrent with comorbidities, tended to smoke, lower BMI, lower socioeconomic status, higher TyG index and TyG-WC index as well as TyG-WHtR index (All *P* < 0.05) when compared with survivors (Table 1). The TyG-related indices were highly correlated with the levels of FLI and BMI (Fig. S3).

**Table 1:** The demographic characteristics of adults with MASLD in the present study

Variables	Total (*n* = 8208)	Survivors (*n* = 6985, 89.0%)	Non-survivors (*n* = 1223, 11.0%)	*P*
TyG, M (Q_1_, Q_3_)	8.96 (8.58, 9.35)	8.94 (8.56, 9.33)	9.11 (8.73, 9.52)	**< 0.001**
TyG-WC, M (Q_1_, Q_3_)	988.27 (921.98, 1083.95)	984.05 (920.04, 1078.81)	1018.51 (951.91, 1117.34)	**< 0.001**
TyG-WHtR, M (Q_1_, Q_3_)	5.86 (5.42, 6.44)	5.83 (5.40, 6.40)	6.09 (5.63, 6.61)	**< 0.001**
Age, M (Q_1_, Q_3_)	49.00 (37.00, 61.00)	47.00 (35.00, 59.00)	67.00 (56.00, 76.00)	**< 0.001**
TC, M (Q_1_, Q_3_)	5.07 (4.40, 5.79)	5.07 (4.42, 5.79)	4.97 (4.27, 5.84)	0.064
ALT, M (Q_1_, Q_3_)	24.00 (18.00, 33.00)	25.00 (19.00, 34.00)	21.00 (17.00, 28.00)	**< 0.001**
AST, M (Q_1_, Q_3_)	23.00 (19.00, 28.00)	23.00 (19.00, 28.00)	23.00 (19.00, 28.00)	0.764
FLI, M (Q_1_, Q_3_)	85.73 (73.61, 94.78)	85.62 (73.48, 94.78)	86.43 (75.56, 94.74)	0.114
BMI, M (Q_1_, Q_3_)	33.18 (30.12, 37.40)	33.30 (30.20, 37.57)	31.99 (29.29, 35.95)	**< 0.001**
Energy intake, M (Q_1_, Q_3_)	2006.00 (1518.25, 2671.59)	2048.17 (1554.00, 2722.00)	1774.40 (1332.62, 2320.74)	**< 0.001**
Gender, n (%)				**0.001**
Male	4209 (54.1)	3470 (53.1)	739 (60.2)	
Female	3999 (45.9)	3515 (46.9)	484 (39.8)	
Race, n (%)				**< 0.001**
Hispanic	2515 (15.8)	2251 (16.6)	264 (7.5)	
Non-Hispanic White	3488 (68.1)	2791 (67.1)	697 (78.2)	
Non-Hispanic Black	1720 (11.2)	1489 (11.2)	231 (10.8)	
Others	485 (4.9)	454 (5.1)	31 (3.5)	
Education level, n (%)				**< 0.001**
≤ High school	4592 (45.7)	3765 (44.8)	827 (60.0)	
College	2282 (32.2)	2033 (32.1)	249 (24.9)	
> College	1334 (22.1)	1187 (23.1)	147 (15.1)	
Marital status, n (%)				**0.043**
Not married	3232 (34.2)	2700 (34.2)	532 (38.1)	
Married/living with partner	4976 (65.8)	4285 (65.8)	691 (61.9)	
PIR, n (%)				**< 0.001**
< 1.3	2506 (21.2)	2086 (20.1)	420 (26.1)	
1.3–3.5	3654 (42.1)	3063 (42.2)	591 (48.8)	
> 3.5	2048 (36.7)	1836 (37.7)	212 (25.1)	
Smoking, n (%)				**< 0.001**
Never	4445 (53.0)	3972 (54.6)	473 (35.7)	
Now	1449 (18.2)	1244 (18.2)	205 (22.3)	
Ever	2314 (28.8)	1769 (27.2)	545 (42.0)	
Alcohol use, n (%)				**< 0.001**
Never	1912 (20.2)	1462 (18.1)	450 (35.4)	
Now	4614 (63.0)	4087 (65.3)	527 (46.3)	
Ever	1682 (16.8)	1436 (16.6)	246 (18.3)	
CVD, n (%)				**< 0.001**
No	7295 (90.7)	6435 (93.1)	860 (71.2)	
Yes	913 (9.3)	550 (6.9)	363 (28.8)	
CKD, n (%)				**< 0.001**
No	7907 (97.1)	6771 (97.5)	1136 (94.2)	
Yes	301 (2.9)	214 (2.5)	87 (5.8)	
Cancer, n (%)				**< 0.001**
No	7423 (90.2)	6441 (91.5)	982 (80.0)	
Yes	785 (9.8)	544 (8.5)	241 (20.0)	
DM, n (%)				**< 0.001**
No	6461 (82.1)	5668 (84.2)	793 (68.1)	
Yes	1747 (17.9)	1317 (15.8)	430 (31.9)	

M, median; Q, quartile; TyG, triglyceride–glucose; WC, waist circumference; WHtR, waist to height ratio; TC, total cholesterol; ALT, glutamic-pyruvic transaminase; AST, and aspartate transaminase; FLI, fatty liver index; BMI, body mass index; PIR, poverty income ratio; CVD, cardiovascular disease; CKD, chronic kidney disease; DM, diabetes mellitus

Non-normally distributed variables are displayed as median with 1st and 3rd quartile (M, Q_1_, Q_3_). Categorical variables are displayed as numbers with weighted percentages (n, %).

Bold values indicate statistical significance (p<0.05)

### Association between TyG index with mortality outcomes of adults with MASLD

**Fig. 2:**
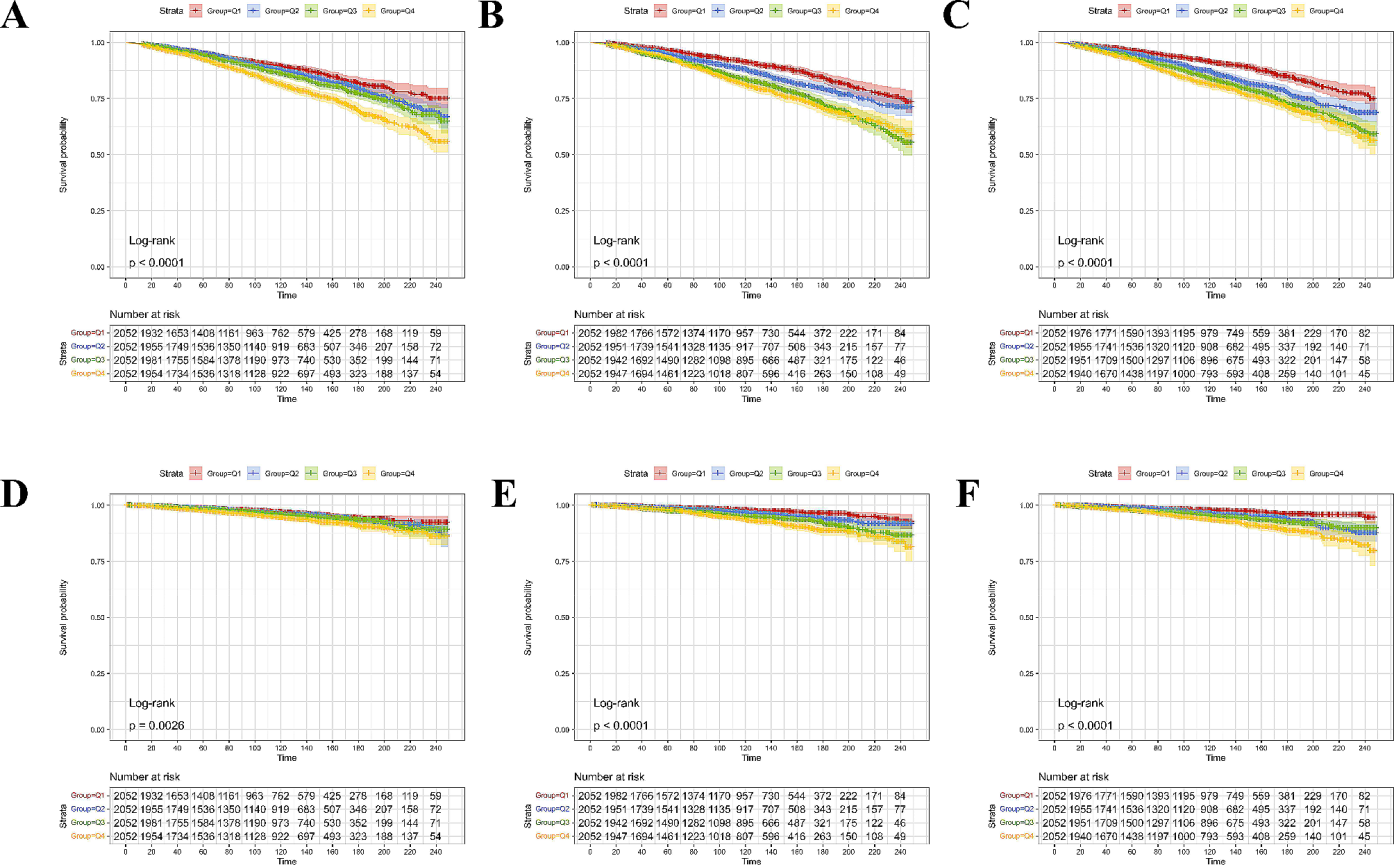
Kaplan–Meier curves show the survival patterns of MASLD adults with different quartile levels of TyG-related indices. **A**–**C** refers to the all-cause mortality of MASLD adults with different quartile levels of TyG-related indices. **D**–**F** refers to the cardiovascular mortality of MASLD adults with different quartile levels of TyG-related indices. TyG, triglyceride–glucose; MASLD, metabolic dysfunction-associated steatotic liver disease; Q, quartile

**Fig. 3:**
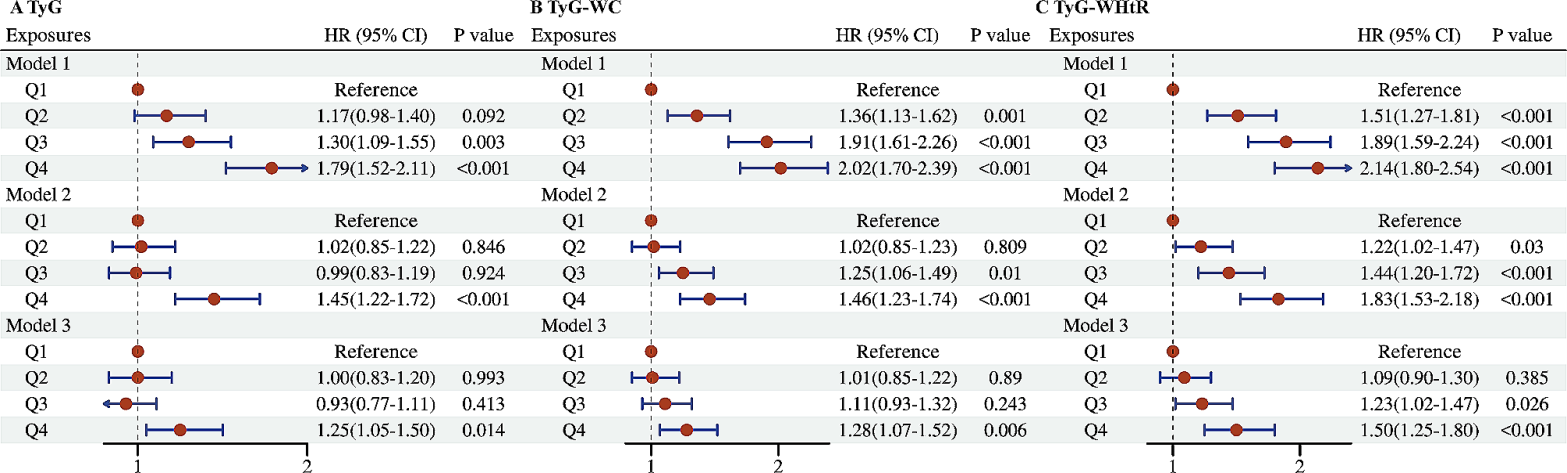
Forest plots show the association between TyG-related indices with the all-cause mortality among adults with MASLD. **A** TyG index; **B** TyG-WC index; **C** TyG-WHtR index. MASLD, metabolic dysfunction-associated steatotic liver disease; TyG, triglyceride–glucose; WC, waist circumference; WHtR, waist to height ratio; Q, quartile. Model 1: unadjusted; Model 2: adjusted for age, gender, race; Model 3: adjusted for age, gender, race, marital status, educational level, energy intakes, poverty income ratio, smoking status, alcohol use, CVD, CKD, cancer, AST, ALT, and TC

**Fig. 4:**
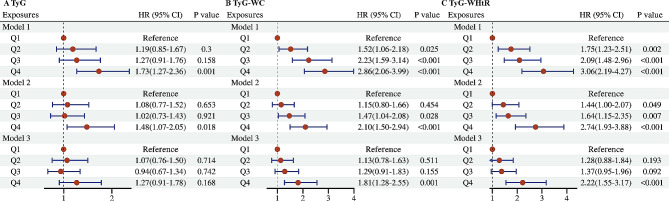
Forest plots show the association between TyG-related indices the cardiovascular mortality among adults with MASLD. **A** TyG index; **B** TyG-WC index; **C** TyG-WHtR index. MASLD, metabolic dysfunction-associated steatotic liver disease; TyG, triglyceride–glucose; WC, waist circumference; WHtR, waist to height ratio; Q, quartile. Model 1: unadjusted; Model 2: adjusted for age, gender, race; Model 3: adjusted for age, gender, race, marital status, educational level, energy intakes, poverty income ratio, smoking status, alcohol use, CVD, CKD, cancer, AST, ALT, and TC

The all-cause mortality was significantly higher in MASLD participants with high quartile levels of TyG, TyG-WC, and TyG-WHtR indices (all *P* < 0.01 by log-rank test) when compared with low quartile subgroups (Fig. 2A, C). Consistently, participants with high levels of TyG-WC index or TyG-WHtR index presented lower cardiovascular-specific survival probabilities when compared with other subgroups (Fig. 2D, F). The multivariate-adjusted Cox regression analysis revealed that the 4th quartile level of the TyG index was significantly associated with the all-cause mortality [adjusted hazard ratio (aHR) = 1.25, 95% confidence interval (CI) 1.05–1.50, *P* = 0.014]. High quartile levels of TyG-WC and TyG-WHtR indices were not only significantly associated with all-cause mortality but also cardiovascular mortality among adults with MASLD (_TyG-WC_aHR for all-cause mortality = 1.28, 95% CI 1.07–1.52, *P* = 0.006; _TyG-WHtR_aHR for all-cause mortality = 1.50, 95% CI 1.25–1.80, *P* < 0.001; _TyG-WC_aHR for cardiovascular mortality = 1.81, 95% CI 1.28–2.55, *P* = 0.001; _TyG-WHtR_aHR for cardiovascular mortality = 2.22, 95% CI 1.55–3.17, *P* < 0.001) (Figs. 3 and 4). The C-index for TyG-related indices in predicting all-cause mortality of MASLD was 0.563 for the TyG index, 0.579 for the TyG-WC index, and 0.585 for the TyG-WHtR index. Similarly, the C-index for TyG-related indices in predicting cardiovascular mortality of MASLD was 0.561 for the TyG index, 0.607 for the TyG-WC index, and 0.615 for the TyG-WHtR index. When combined with other clinical factors, the C-index was 0.819 for the TyG index, 0.824 for the TyG-WC index, and 0.830 for the TyG-WHtR index in predicting all-cause mortality and the C-index was 0.829 for the TyG index, 0.838 for the TyG-WC index, and 0.844 for the TyG-WHtR index in predicting cardiovascular mortality among participants with MASLD.

### The non-linear trend of TyG-related indices with mortality outcomes of adults with MASLD

**Fig. 5:**
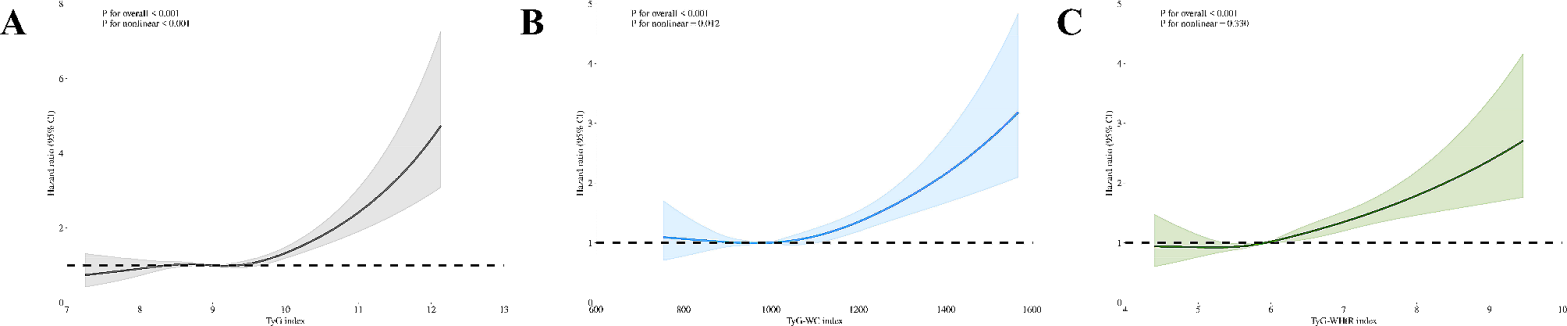
Restricted cubic splines reflect the dose-effect relationships between TyG-related indices with the all-cause mortality among adults with MASLD. **A** TyG index; **B** TyG-WC index; **C** TyG-WHtR index. MASLD, metabolic dysfunction-associated steatotic liver disease; TyG, triglyceride–glucose; WC, waist circumference; WHtR, waist to height ratio

**Fig. 6:**
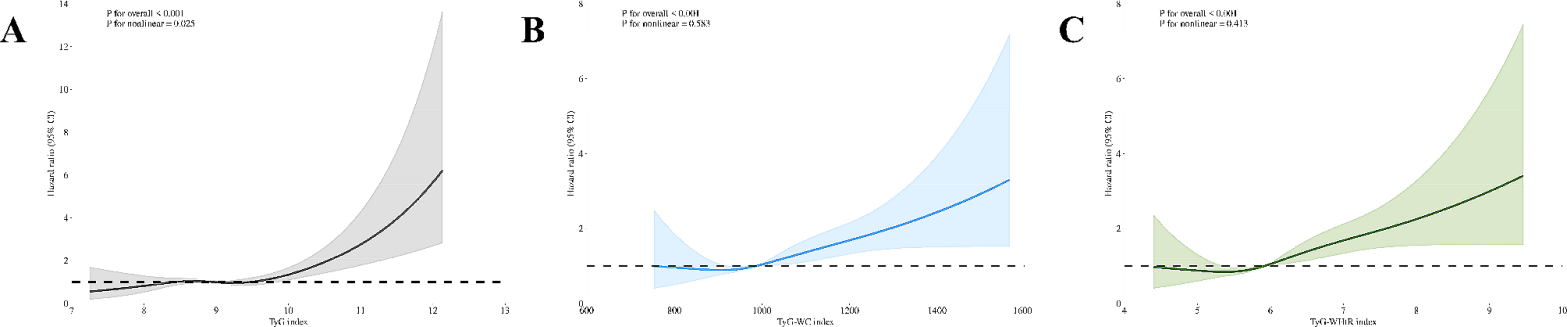
Restricted cubic splines reflect the dose-effect relationships between TyG-related indices the cardiovascular mortality among adults with MASLD. **A** TyG index; **B** TyG-WC index; **C** TyG-WHtR index. MASLD, metabolic dysfunction-associated steatotic liver disease; TyG, triglyceride–glucose; WC, waist circumference; WHtR, waist to height ratio

The multivariate-adjusted RCS plots revealed a non-linear trend between TyG and TyG-WC indices with all-cause mortality of participants with MASLD (P for non-linear < 0.001 and = 0.012, respectively) but a linear trend was observed between TyG-WHtR index with all-cause mortality of adults with MASLD (P for overall < 0.001) (Fig. 5A–C). Additionally, only the TyG index showed non-linearity with the cardiovascular mortality of participants with MASLD (P for non-linear = 0.025) (Fig. 6A). By contrast, linear trends were observed between TyG-WC and TyG-WHtR indices with the cardiovascular mortality of participants with MASLD (P for overall < 0.001) (Fig. 6B, C).

### Subgroup and sensitive analyses

The subgroup analyses revealed a heightened predictive significance of TyG-related indices in specific demographic groups: individuals aged < 65 years old, males (notably observed in the TyG-WHtR index), those of Hispanic ethnicity (especially notable in the TyG-WC and TyG-WHtR indices), individuals with lower BMI (particularly evident in the TyG-WHtR index), unmarried individuals (particularly evident in the TyG-WHtR index), and those without comorbidities (Figs. S4–S6). Some interactions were observed between the TyG-related indices with other covariates, including gender, age, marital status, history of comorbidities, and living habits. Meanwhile, we conducted a series of sensitive analyses to check the robustness of the primary findings. First, consistent associations between TyG-related indices with all-cause as well as cardiovascular mortality were observed after excluding the participants who died within 2 years (Tables S1–S3). Additionally, among the adults in the earlier cycles of the interview, TyG-related indices still showed significant associations with the mortality outcomes of adults with MASLD (Tables S4–S6). Last, the TyG-related indices showed similar associations with the mortality outcomes of adults with more loose inclusion criteria for the diagnosis of SLD (FLI ≥ 30) (Tables S7–S9).

## Discussion

In the current study, we determined that elevated levels of TyG-related indices were significantly associated with the mortality outcomes of adults with MASLD. Specifically, the MASLD participants with the 4th quartile levels of TyG, TyG-WC, and TyG-WHtR indices showed approximately 1.25-, 1.28-, and 1.50-fold increased risks of all-cause mortality, respectively. Besides, TyG-WC and TyG-WHtR indices exhibited optimal predictive power in cardiovascular mortality of participants with MASLD, with a C-index of 0.607 and 0.615, respectively. Moreover, non-linearity was observed between the TyG index with the all-cause and cardiovascular mortality of participants with MASLD. To the best of our knowledge, this is the first study to evaluate the prognostic effect and dose-effect relationships between the TyG-related indices with the mortality risks among US adults with MASLD. Our findings indicate that TyG-related indices may be surrogate prognostic biomarkers for the clinical management of MASLD.

To date, the strong epidemiological and pathogenic associations between NAFLD, metabolic syndrome (MetS), and IR requested the name change for NAFLD. Especially, the Delphi consensus statement introduced a new nomenclature (MASLD) and diagnostic criteria to replace the term NAFLD as a means to improve awareness and patient identification without stigma [7]. The latest evidence demonstrated similar clinical profiles and mortality rates between MASLD and NAFLD, which validated the utility of MASLD in future clinical practice [46]. Notably, among the varied cardiometabolic risk factors, IR has been regarded as the cornerstone of occurrence as well as the progress of MASLD [47]. In the IR condition, the inhibition of lipolysis is impaired, resulting in increased serum levels of free fatty acids (FFAs), which ultimately increase the lipids accumulation in the liver. Meanwhile, IR could also enhance gluconeogenesis but decrease hepatic glycogenesis, which can increase glucose production and release [48, 49]. Therefore, the tight association between IR and MASLD indicated that IR-related biomarkers would bring certain benefits for the early detection of MASLD and the complications risk prediction [50].

TyG index, as one simple, readily available, and easily measurable parameter for detecting IR, has been observed to be associated with the onset and prognosis of numerous cardiometabolic-related diseases [51, 52]. In particular, the TyG index maintained high predictive accuracy in predicting the development of CVD and cardiovascular mortality in adults with diabetes or pre-diabetes [51, 53]. Similarly, among adults with metabolic syndrome, Wei et al. determined that the TyG index was significantly correlated with the diabetic mortality of this population [54]. Consistently, the TyG index was also associated with the onset and progress of NAFLD. Of note, Zhang et al. determined that the TyG index presented optimal clinical value in identifying high-risk individuals for NAFLD, with good predictive accuracy [55]. Moreover, Zhao et al. demonstrated that high levels of TyG index were not only associated with increased risks of coronary heart disease but related to the severity of coronary atherosclerosis in patients with NAFLD [50]. Similarly, the promising risk prediction value of the TyG index in NAFLD was also observed in other countries and regions [56–58]. Although the positive relationships between the TyG index and MASLD have been determined, the prognostic effect of the TyG index on this population remained unclear [23, 59]. In this study, we filled this research gap and took it further based on one representative nationwide cohort. Specifically, the 4th quartile of the TyG index was independently associated with increased all-cause mortality of participants with MASLD in the current study, with adjustment for a series of demographic characteristics. The RCS curves revealed a non-linear trend between the TyG index with all-cause and cardiovascular mortality risks of MASLD, which indicated the value of 8.9 would be a potential cutoff point for risk stratification. Mechanically, the TyG index reflected the peripheral blood glucose and lipid profiles and was closely associated with host systemic inflammation, oxidative stress, and endothelial dysfunction [60, 61]. The disorders in glucose and lipid metabolism, reflected in the elevated TyG index, thereby might be an early sign of adverse events in patients with MASLD [50]. Summarily, our study, along with previous works, highlighted the prognostic effect of the TyG index on the prognosis of adults with MASLD. However, the moderate predictive accuracy of the TyG index prompted us to investigate additional adjusted TyG-related indices for predicting the mortality outcomes of adults with MASLD.

Notably, strong evidence has demonstrated that WC and WHtR were simple and useful tools to reflect the central obesity of the population and are significantly associated with all-cause and CVD mortality [62, 63]. Therefore, TyG index-related parameters including TyG-WC and TyG-WHtR indices were recently proposed, which have been determined to exhibit improved predictive accuracy than the original TyG index in identifying the occurrence and mortality risks of cardiometabolic-related diseases [24, 26]. In this study, the TyG-WC and TyG-WHtR indices were found to be more closely associated with levels of FLI and BMI compared to the original TyG index, which indicated the optimal role of TyG-WC and TyG-WHtR indices in reflecting lipid metabolism disorders in MASLD. Consistently, the findings of this study demonstrated that the TyG-WC and TyG-WHtR indices provided independent predictive value for survival in MASLD. Specifically, the C-index of TyG-WC and TyG-WHtR indices reached approximately 0.83 for predicting all-cause mortality and 0.84 for predicting cardiovascular mortality, respectively, when combined with other prognostic factors. Therefore, our findings, along with results from previous studies, suggest that adiposity-adjusted TyG indices could serve as effective tools to assist clinicians in making tailored mortality risk predictions for adults with MASLD.

The stratified analyses revealed that the prediction value of TyG-related indices varied among different subpopulations. Especially, younger and middle-aged adults (< 65 years) with MASLD were more sensitive to the prognostic effects of TyG-related indices with comparison to those at older ages. In line with our findings, one large-scale retrospective study revealed that the TyG-related indices presented superior DM detection ability in the younger and middle-aged populations [64]. Similarly, among the general population, adults of younger age (< 50 years) presented significantly higher risk for CVD than older adults at the same levels of TyG index [65]. Consequently, the potential benefits of monitoring TyG-related indices for the prevention of metabolic-related diseases and the reduction of survival risks appear to be more pronounced in younger adults. Future large-scale epidemiological studies regarding the associations between TyG-related indices and the prognosis of MASLD in younger adults should be conducted, which may have the potential to provide more accurate information essential for personalized prognosis assessment.

### Strength and limitation

There are some strengths in this work. First, this is a pilot study, to our knowledge, to explore the prognostic effect of TyG-related indices on mortality outcomes among US adults with MASLD. Our findings highlight the clinical value of TyG-related indices, particularly the TyG-WHtR index, in the management of the MASLD population. Second, the study sample size is promising and the follow-up time is sufficient to observe the mortality outcomes. Additionally, we have controlled a series of covariates to determine the independent associations between TyG-related indices with the mortality outcomes of MASLD. Last, we conduct several sensitive analyses to validate the robustness of the main findings.

Some limitations need to be addressed in the future study. First, the diagnosis of SLD was established using FLI ≥ 60 instead of histological or ultrasonographic assessment. While the FLI may offer an advantage by primarily selecting patients with MASLD [30], it is not the optimal tool to distinguish the specific causes of SLD. Second, although we controlled a series of demographic and socioeconomic associated factors during the analysis, some residual covariates such as healthy eating patterns which were not available in the whole study population, were not further controlled. To reduce the potential influence of dietary on the metabolism of adults, we applied the total energy intake as the covariate to refer to the dietary patterns of the individuals. Furthermore, the TyG-related indices were calculated by using the baseline data, which cannot allow us to assess the longitudinal changes of TyG-related indices with the clinical outcomes of MASLD over time. Last, the results were derived from the US population, whether the findings could be applied to other regions with different races are worth exploring. Future prospective longitudinal works are warranted to determine the optimal cutoff value and provide stronger evidence to support the clinical application of TyG-related indices in the follow-up management of adults with MASLD.

## Conclusion

In conclusion, our findings suggest that elevated levels of TyG-related indices are correlated with increased risks of all-cause and cardiovascular mortality in US adults with MASLD. Furthermore, compared to the original TyG index, the TyG-WHtR index demonstrates an enhanced prognostic effect on mortality outcomes in adults with MASLD, making it a simple and easily calculable clinical biomarker for managing MASLD. Future well-designed, longitudinal, prospective studies are warranted to validate our findings.

### Electronic supplementary material

Below is the link to the electronic supplementary material.

Supplementary Material 1.

## Data Availability

The datasets generated during and/or analyzed during the current study are available from the corresponding author upon reasonable request.
