# Supplementary material for: Prognostic effect of triglyceride glucose-related parameters on all-cause and cardiovascular mortality in the United States adults with metabolic dysfunction-associated steatotic liver disease
Source: Cardiovasc Diabetol. 2024 Jun 1;23:188. doi: 10.1186/s12933-024-02287-y (PMC11144336; doi:10.1186/s12933-024-02287-y)
Supplement: Supplementary file 1 — Supplementary Material 1. [file 12933_2024_2287_MOESM1_ESM.docx]

**Supporting information**

**Prognostic effect of triglyceride glucose-related parameters on all-cause and cardiovascular mortality in the United States adults with metabolic dysfunction-associated steatotic liver disease**

**Figure S1**. The distribution of the continuous variables in this study. TyG: triglyceride-glucose; WC: waist circumference; WHtR: waist to height ratio; BMI: body mass index; TC: total cholesterol; ALT: glutamic-pyruvic transaminase; AST: and aspartate transaminase; FLI: fatty liver index.


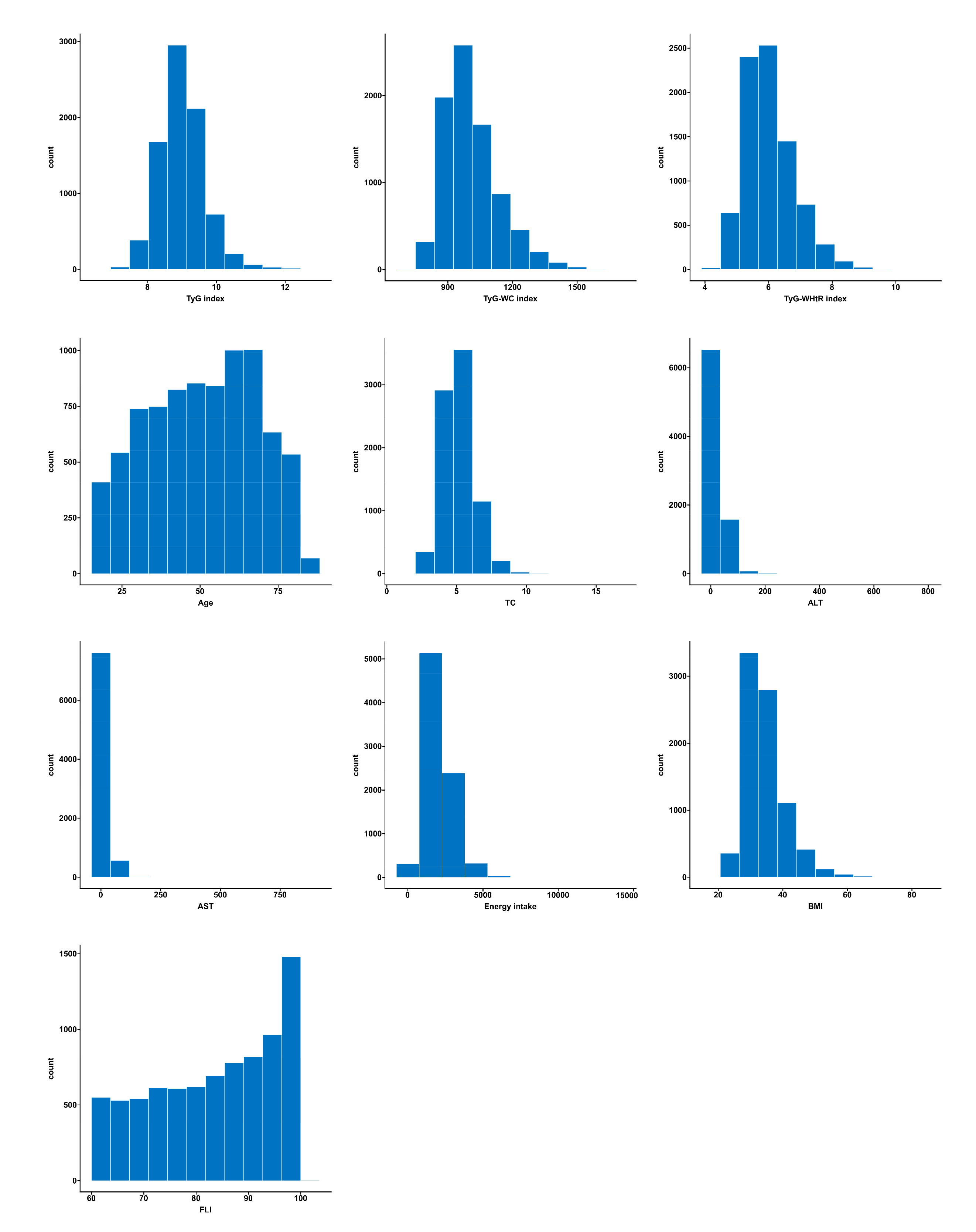


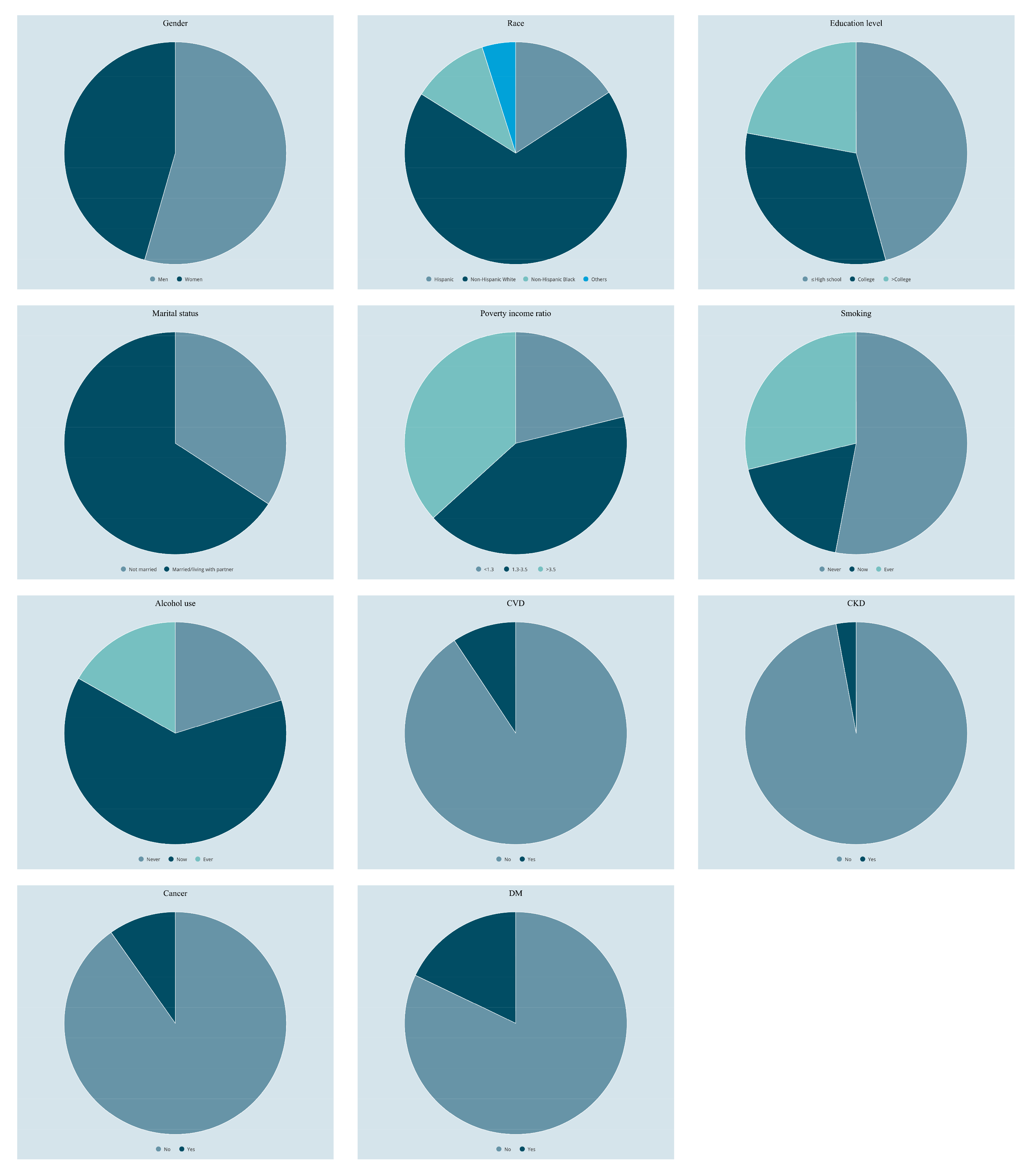
**Figure S2.** The weighted percentage of categorical variables in this study. The proportion of the pie chart refers to the weighted percentage of the demographic features. CVD: cardiovascular disease; CKD: chronic kidney disease; DM: diabetes mellitus.

**Figure S3**. The correlations between TyG-related indices with other variables. TyG: triglyceride-glucose; WC: waist circumference; WHtR: waist to height ratio; BMI: body mass index; FLI: fatty liver index. The proportion of the pie chart refers to the Pearson correlation coefficient (from 0 to 1). ^**^Two-tail Pearson test p<0.01, ^***^Two-tail Pearson test p<0.001.


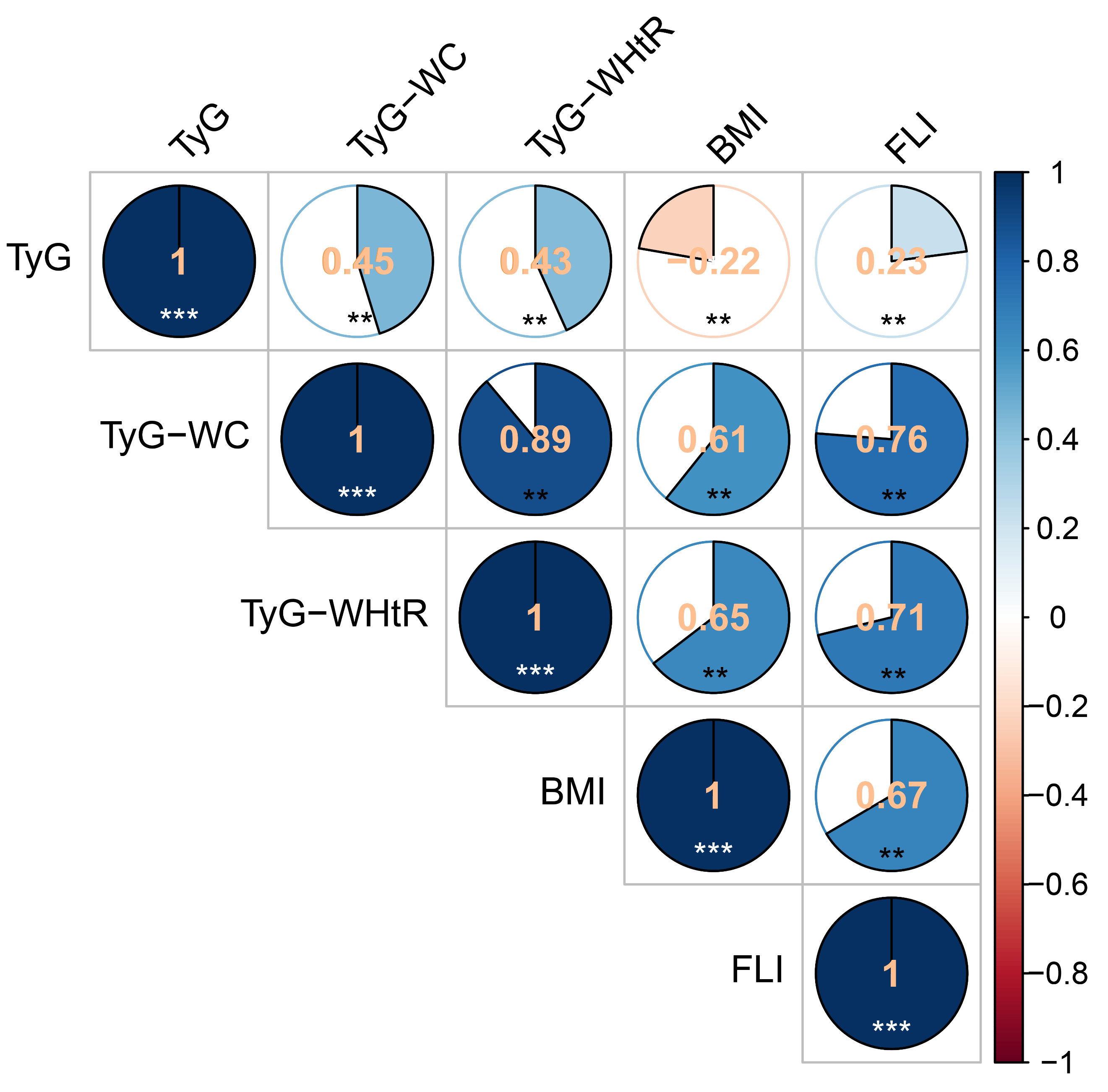


**Figure S4**. Association between TyG index with all-cause mortality among adults with MASLD in different subgroups. TyG: triglyceride-glucose; BMI: body mass index; PIR: poverty income ratio; CVD: cardiovascular disease; CKD: chronic kidney disease; DM: diabetes mellitus


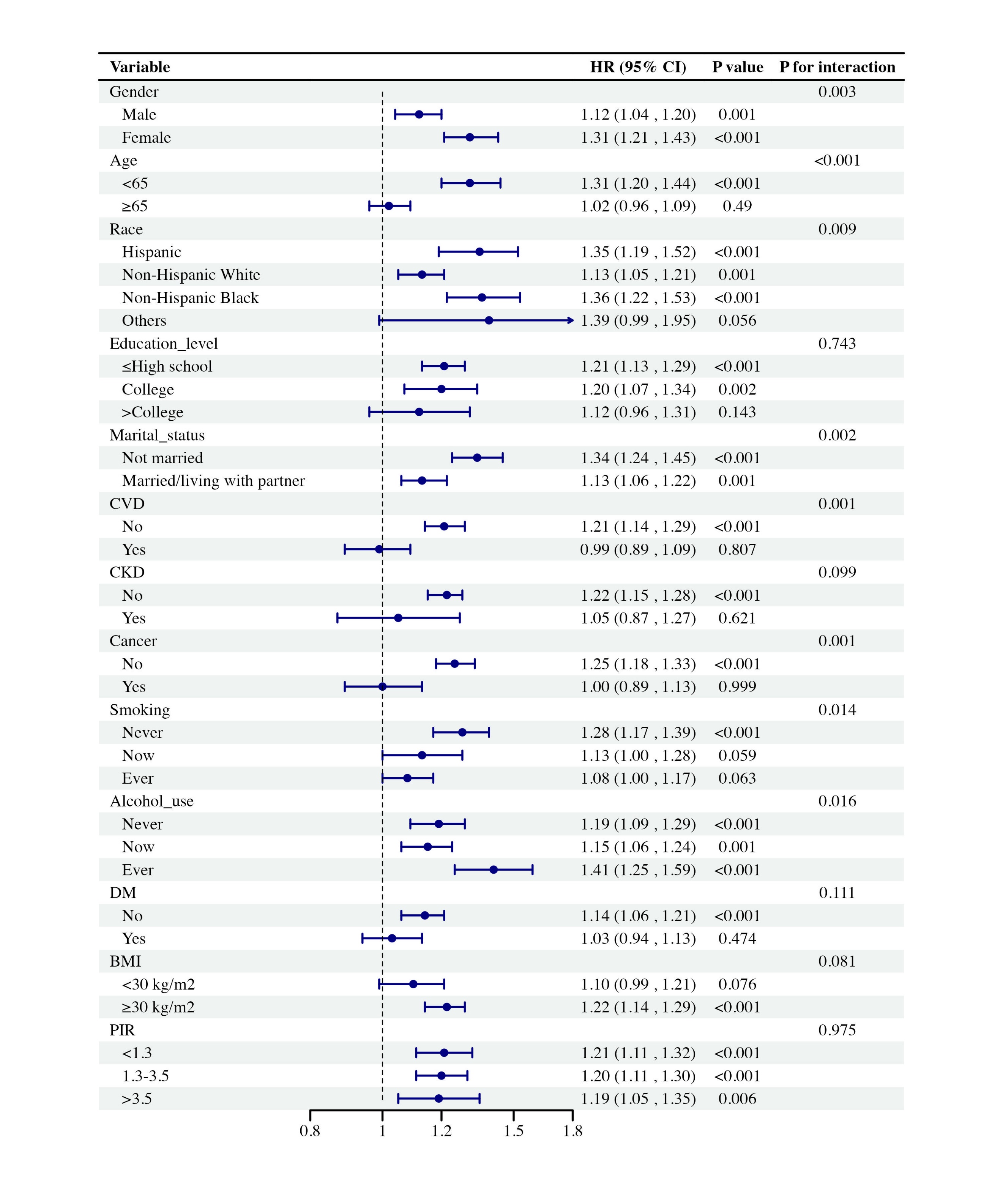


**Figure S5**. Association between TyG-WC index with all-cause mortality among adults with MASLD in different subgroups. TyG: triglyceride-glucose; WC: waist circumference; BMI: body mass index; PIR: poverty income ratio; CVD: cardiovascular disease; CKD: chronic kidney disease; DM: diabetes mellitus


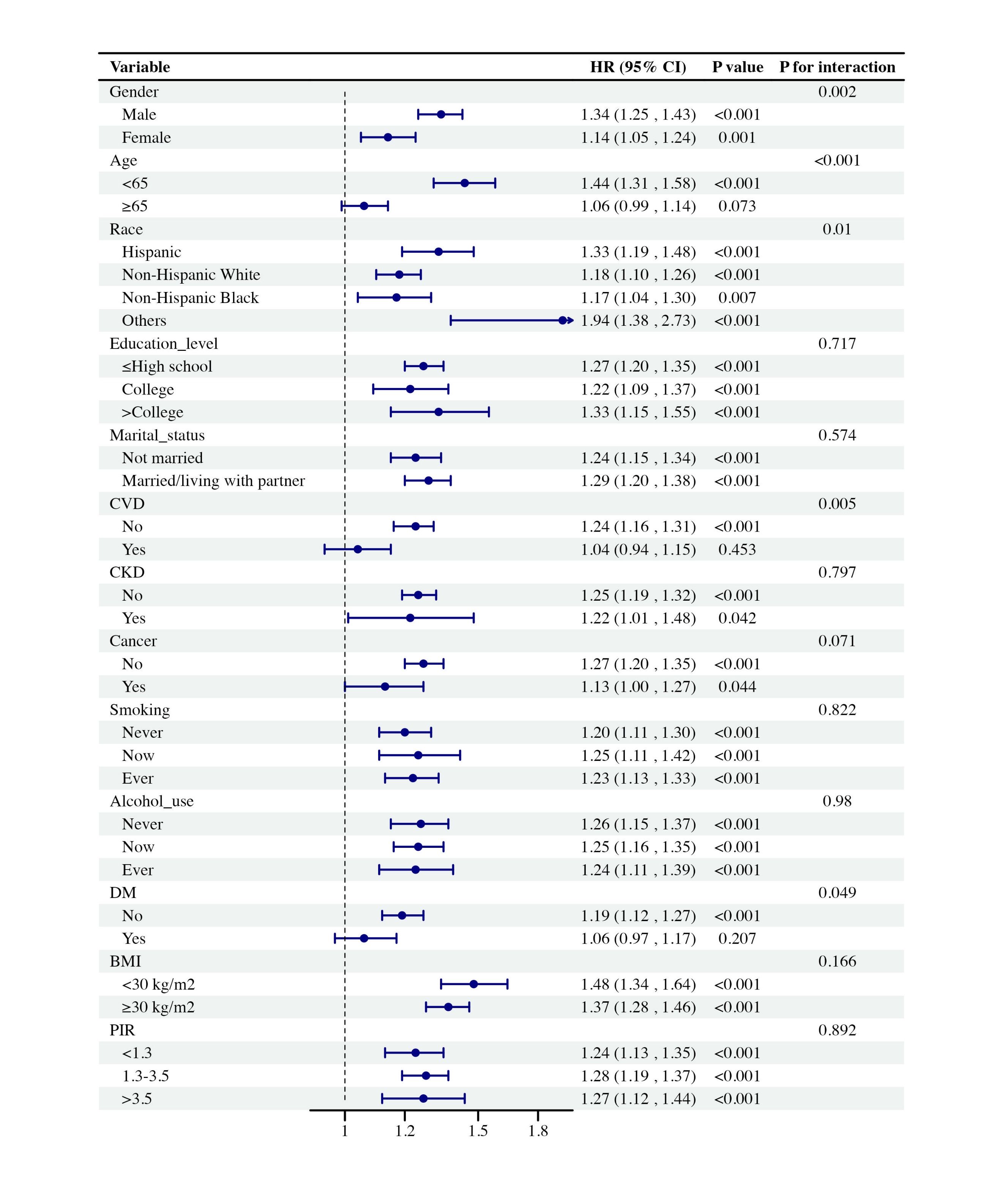


**Figure S6**. Association between TyG-WHtR index with all-cause mortality among adults with MASLD in different subgroups. TyG: triglyceride-glucose; WHtR: waist to height ratio; BMI: body mass index; PIR: poverty income ratio; CVD: cardiovascular disease; CKD: chronic kidney disease; DM: diabetes mellitus


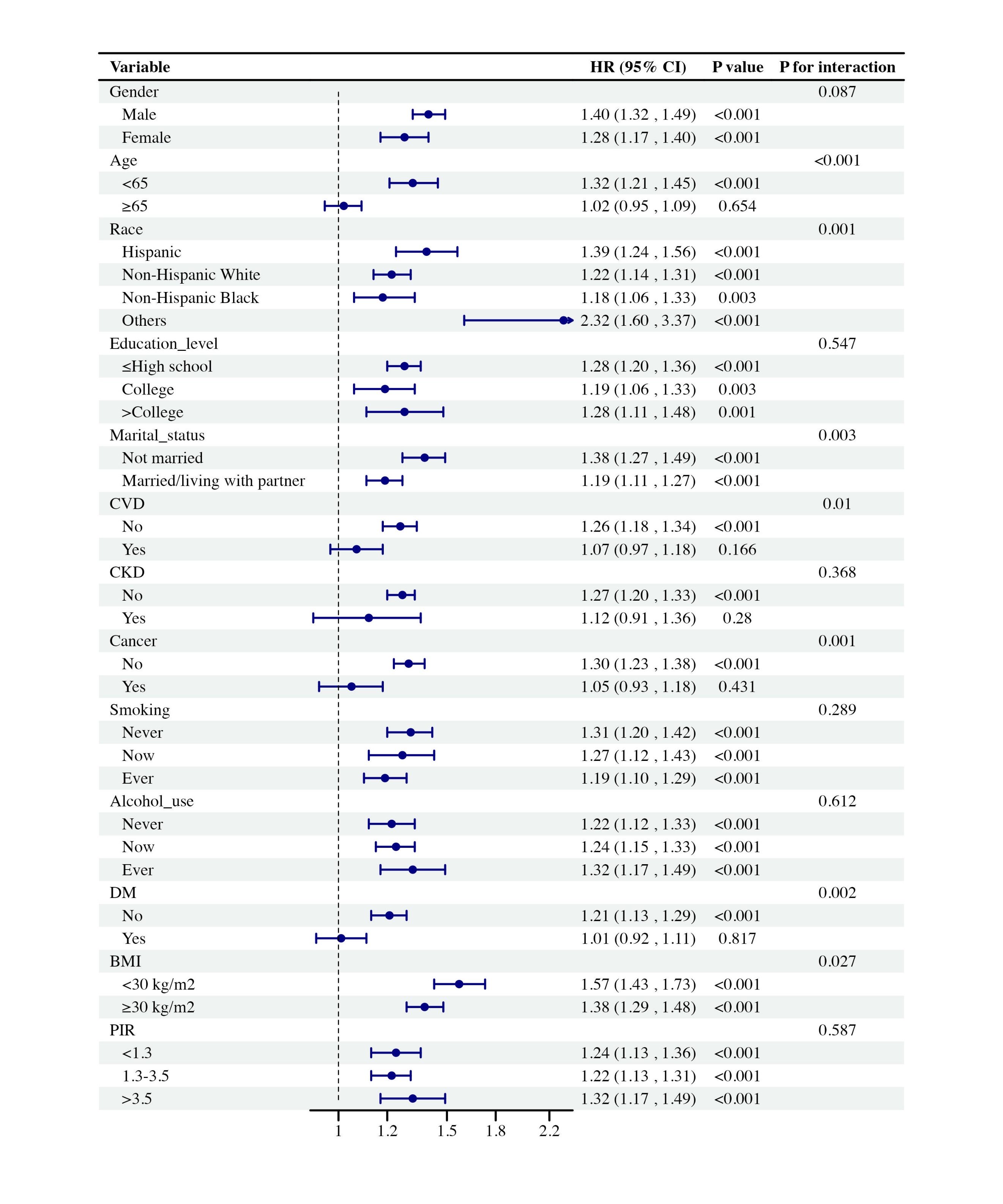


**Table S1**. Sensitive analysis to evaluate the association between the TyG index with the mortality outcomes of adults with MASLD by excluding participants who died within 2 years.

| **Subgroup** | **Model 1** | | **Model 2** | | **Model 3** | |
| --- | --- | --- | --- | --- | --- | --- |
|  | **HR (95% CI)** | **P** | **HR (95% CI)** | **P** | **HR (95% CI)** | **P** |
| **All-cause mortality** | | | | | | |
| Q1 | Reference | | Reference | | Reference | |
| Q2 | 1.22(1.00-1.48) | **0.048** | 1.06(0.87-1.28) | 0.587 | 1.03(0.84-1.25) | 0.787 |
| Q3 | 1.35(1.12-1.63) | **0.002** | 1.03(0.85-1.25) | 0.785 | 0.95(0.78-1.16) | 0.618 |
| Q4 | 1.85(1.54-2.21) | **<.001** | 1.49(1.23-1.79) | **<.001** | 1.29(1.06-1.56) | **0.012** |
| **Cardiovascular mortality** | | | | | | |
| Q1 | Reference | | Reference | | Reference | |
| Q2 | 1.26(0.87-1.81) | 0.219 | 1.15(0.80-1.67) | 0.447 | 1.14(0.79-1.66) | 0.482 |
| Q3 | 1.35(0.94-1.92) | 0.104 | 1.11(0.77-1.60) | 0.588 | 1.04(0.71-1.51) | 0.859 |
| Q4 | 1.81(1.29-2.55) | **0.001** | 1.60(1.12-2.28) | **0.010** | 1.39(0.96-2.01) | 0.082 |

Abbreviation: TyG: triglyceride-glucose; HR: hazard ratio; CI: confidence interval; Q: quartile.

Model 1: unadjusted; Model 2: adjusted for age, gender, race; Model 3: adjusted for age, gender, race, marital status, educational level, energy intakes, poverty income ratio, smoking status, alcohol use, CVD, CKD, cancer, AST, ALT, and TC.

**Table S2**. Sensitive analysis to evaluate the association between the TyG-WC index with the mortality outcomes of adults with MASLD by excluding participants who died within 2 years.

| **Subgroup** | **Model 1** | | **Model 2** | | **Model 3** | |
| --- | --- | --- | --- | --- | --- | --- |
|  | **HR (95% CI)** | **P** | **HR (95% CI)** | **P** | **HR (95% CI)** | **P** |
| **All-cause mortality** | | | | | | |
| Q1 | Reference | | Reference | | Reference | |
| Q2 | 1.28(1.06-1.57) | **0.011** | 0.97(0.80-1.17) | 0.723 | 0.96(0.79-1.16) | 0.654 |
| Q3 | 1.91(1.60-2.28) | **<.001** | 1.25(1.04-1.50) | **0.016** | 1.11(0.92-1.33) | 0.276 |
| Q4 | 1.99(1.66-2.38) | **<.001** | 1.45(1.20-1.74) | **<.001** | 1.28(1.06-1.54) | **0.010** |
| **Cardiovascular mortality** | | | | | | |
| Q1 | Reference | | Reference | | Reference | |
| Q2 | 1.46(0.99-2.15) | 0.055 | 1.11(0.75-1.64) | 0.601 | 1.09(0.74-1.61) | 0.668 |
| Q3 | 2.14(1.49-3.08) | **<.001** | 1.41(0.98-2.04) | 0.067 | 1.24(0.86-1.80) | 0.257 |
| Q4 | 2.83(1.99-4.02) | **<.001** | 2.08(1.46-2.97) | **<.001** | 1.80(1.25-2.59) | **0.002** |

Abbreviation: TyG: triglyceride-glucose; WC: waist circumference; HR: hazard ratio; CI: confidence interval; Q: quartile.

Model 1: unadjusted; Model 2: adjusted for age, gender, race; Model 3: adjusted for age, gender, race, marital status, educational level, energy intakes, poverty income ratio, smoking status, alcohol use, CVD, CKD, cancer, AST, ALT, and TC.

**Table S3**. Sensitive analysis to evaluate the association between TyG-WHtR index with the mortality outcomes of adults with MASLD by excluding participants who died within 2 years.

| **Subgroup** | **Model 1** | | **Model 2** | | **Model 3** | |
| --- | --- | --- | --- | --- | --- | --- |
|  | **HR (95% CI)** | **P** | **HR (95% CI)** | **P** | **HR (95% CI)** | **P** |
| **All-cause mortality** | | | | | | |
| Q1 | Reference | | Reference | | Reference | |
| Q2 | 1.54(1.28-1.87) | **<.001** | 1.25(1.03-1.52) | **0.024** | 1.11(0.91-1.35) | 0.290 |
| Q3 | 1.91(1.59-2.29) | **<.001** | 1.45(1.20-1.76) | **<.001** | 1.25(1.03-1.51) | **0.024** |
| Q4 | 2.16(1.80-2.60) | **<.001** | 1.85(1.53-2.24) | **<.001** | 1.54(1.27-1.87) | **<.001** |
| **Cardiovascular mortality** | | | | | | |
| Q1 | Reference | | Reference | | Reference | |
| Q2 | 1.75(1.20-2.56) | **0.004** | 1.45(0.98-2.13) | 0.061 | 1.29(0.87-1.90) | 0.203 |
| Q3 | 1.95(1.34-2.83) | **<.001** | 1.54(1.05-2.27) | **0.027** | 1.29(0.88-1.91) | 0.198 |
| Q4 | 3.05(2.14-4.34) | **<.001** | 2.77(1.91-4.00) | **<.001** | 2.28(1.56-3.33) | **<.001** |

Abbreviation: TyG: triglyceride-glucose; WHtR: waist to height ratio; HR: hazard ratio; CI: confidence interval; Q: quartile.

Model 1: unadjusted; Model 2: adjusted for age, gender, race; Model 3: adjusted for age, gender, race, marital status, educational level, energy intakes, poverty income ratio, smoking status, alcohol use, CVD, CKD, cancer, AST, ALT, and TC.

**Table S4**. Sensitive analysis to evaluate the association between the TyG index with the mortality outcomes of adults with MASLD by only including participants from 1999 to 2006.

| **Subgroup** | **Model 1** | | **Model 2** | | **Model 3** | |
| --- | --- | --- | --- | --- | --- | --- |
|  | **HR (95% CI)** | **P** | **HR (95% CI)** | **P** | **HR (95% CI)** | **P** |
| **All-cause mortality** | | | | | | |
| Q1 | Reference | | Reference | | Reference | |
| Q2 | 1.17(0.91-1.50) | 0.217 | 0.99(0.77-1.27) | 0.908 | 0.94(0.73-1.21) | 0.604 |
| Q3 | 1.31(1.03-1.66) | **0.027** | 0.97(0.76-1.24) | 0.820 | 0.87(0.68-1.13) | 0.294 |
| Q4 | 1.97(1.57-2.46) | **<.001** | 1.53(1.21-1.94) | **<.001** | 1.32(1.03-1.69) | **0.027** |
| **Cardiovascular mortality** | | | | | | |
| Q1 | Reference | | Reference | | Reference | |
| Q2 | 1.08(0.67-1.71) | 0.762 | 0.95(0.60-1.53) | 0.846 | 0.92(0.57-1.49) | 0.725 |
| Q3 | 1.26(0.80-1.97) | 0.318 | 1.00(0.63-1.59) | 0.998 | 0.91(0.56-1.47) | 0.699 |
| Q4 | 2.02(1.33-3.06) | **0.001** | 1.70(1.10-2.62) | **0.018** | 1.46(0.92-2.30) | 0.108 |

Abbreviation: TyG: triglyceride-glucose; HR: hazard ratio; CI: confidence interval; Q: quartile.

Model 1: unadjusted; Model 2: adjusted for age, gender, race; Model 3: adjusted for age, gender, race, marital status, educational level, energy intakes, poverty income ratio, smoking status, alcohol use, CVD, CKD, cancer, AST, ALT, and TC.

**Table S5**. Sensitive analysis to evaluate the association between the TyG-WC index with the mortality outcomes of adults with MASLD by only including participants from 1999 to 2006.

| **Subgroup** | **Model 1** | | **Model 2** | | **Model 3** | |
| --- | --- | --- | --- | --- | --- | --- |
|  | **HR (95% CI)** | **P** | **HR (95% CI)** | **P** | **HR (95% CI)** | **P** |
| **All-cause mortality** | | | | | | |
| Q1 | Reference | | Reference | | Reference | |
| Q2 | 1.24(0.98-1.55) | 0.070 | 0.96(0.77-1.21) | 0.744 | 0.95(0.76-1.20) | 0.688 |
| Q3 | 1.70(1.37-2.11) | **<.001** | 1.12(0.90-1.39) | 0.324 | 1.03(0.82-1.29) | 0.797 |
| Q4 | 1.86(1.49-2.31) | **<.001** | 1.42(1.14-1.78) | **0.002** | 1.38(1.10-1.73) | **0.005** |
| **Cardiovascular mortality** | | | | | | |
| Q1 | Reference | | Reference | | Reference | |
| Q2 | 1.25(0.79-2.00) | 0.344 | 0.98(0.62-1.57) | 0.943 | 0.96(0.60-1.54) | 0.860 |
| Q3 | 1.79(1.15-2.78) | **0.010** | 1.18(0.76-1.84) | 0.469 | 1.11(0.70-1.74) | 0.668 |
| Q4 | 3.03(2.00-4.57) | **<.001** | 2.33(1.54-3.53) | **<.001** | 2.33(1.52-3.56) | **<.001** |

Abbreviation: TyG: triglyceride-glucose; WC: waist circumference; HR: hazard ratio; CI: confidence interval; Q: quartile.

Model 1: unadjusted; Model 2: adjusted for age, gender, race; Model 3: adjusted for age, gender, race, marital status, educational level, energy intakes, poverty income ratio, smoking status, alcohol use, CVD, CKD, cancer, AST, ALT, and TC.

**Table S6**. Sensitive analysis to evaluate the association between the TyG-WHtR index with the mortality outcomes of adults with MASLD by only including participants from 1999 to 2006.

| **Subgroup** | **Model 1** | | **Model 2** | | **Model 3** | |
| --- | --- | --- | --- | --- | --- | --- |
|  | **HR (95% CI)** | **P** | **HR (95% CI)** | **P** | **HR (95% CI)** | **P** |
| **All-cause mortality** | | | | | | |
| Q1 | Reference | | Reference | | Reference | |
| Q2 | 1.51(1.20-1.90) | **<.001** | 1.28(1.02-1.62) | **0.036** | 1.17(0.93-1.48) | 0.187 |
| Q3 | 1.77(1.42-2.21) | **<.001** | 1.40(1.11-1.76) | **0.004** | 1.29(1.02-1.62) | **0.035** |
| Q4 | 2.04(1.63-2.55) | **<.001** | 1.84(1.45-2.32) | **<.001** | 1.69(1.33-2.15) | **<.001** |
| **Cardiovascular mortality** | | | | | | |
| Q1 | Reference | | Reference | | Reference | |
| Q2 | 2.06(1.27-3.34) | **0.004** | 1.84(1.12-3.00) | **0.015** | 1.73(1.05-2.85) | **0.031** |
| Q3 | 2.37(1.47-3.80) | **<.001** | 2.01(1.24-3.27) | **0.005** | 1.81(1.10-2.97) | **0.019** |
| Q4 | 3.89(2.47-6.12) | **<.001** | 3.86(2.41-6.18) | **<.001** | 3.71(2.29-6.01) | **<.001** |

Abbreviation: TyG: triglyceride-glucose; WHtR: waist to height ratio; HR: hazard ratio; CI: confidence interval; Q: quartile.

Model 1: unadjusted; Model 2: adjusted for age, gender, race; Model 3: adjusted for age, gender, race, marital status, educational level, energy intakes, poverty income ratio, smoking status, alcohol use, CVD, CKD, cancer, AST, ALT, and TC.

**Table S7**. Sensitive analysis to evaluate the association between TyG index with the mortality outcomes of adults with FLI ≥ 30.

| **Subgroup** | **Model 1** | | **Model 2** | | **Model 3** | |
| --- | --- | --- | --- | --- | --- | --- |
|  | **HR (95% CI)** | **P** | **HR (95% CI)** | **P** | **HR (95% CI)** | **P** |
| **All-cause mortality** | | | | | | |
| Q1 | Reference | | Reference | | Reference | |
| Q2 | 1.40(1.21-1.62) | **<.001** | 1.19(1.03-1.39) | **0.021** | 1.15(0.99-1.34) | 0.065 |
| Q3 | 1.54(1.33-1.78) | **<.001** | 1.23(1.06-1.42) | **0.006** | 1.18(1.01-1.37) | **0.032** |
| Q4 | 1.73(1.68-2.21) | **<.001** | 1.52(1.32-1.75) | **<.001** | 1.38(1.19-1.60) | **<.001** |
| **Cardiovascular mortality** | | | | | | |
| Q1 | Reference | | Reference | | Reference | |
| Q2 | 1.23(0.93-1.63) | 0.142 | 1.09(0.82-1.44) | 0.562 | 1.05(0.79-1.40) | 0.722 |
| Q3 | 1.54(1.18-2.00) | **0.001** | 1.29(0.98-1.69) | 0.066 | 1.25(0.95-1.64) | 0.112 |
| Q4 | 1.75(1.35-2.26) | **<.001** | 1.46(1.12-1.90) | **0.006** | 1.31(0.99-1.73) | 0.053 |

Abbreviation: TyG: triglyceride-glucose; FLI: fatty liver index; HR: hazard ratio; CI: confidence interval; Q: quartile.

Model 1: unadjusted; Model 2: adjusted for age, gender, race; Model 3: adjusted for age, gender, race, marital status, educational level, energy intakes, poverty income ratio, smoking status, alcohol use, CVD, CKD, cancer, AST, ALT, and TC.

**Table S8**. Sensitive analysis to evaluate the association between TyG-WC index with the mortality outcomes of adults with FLI ≥ 30.

| **Subgroup** | **Model 1** | | **Model 2** | | **Model 3** | |
| --- | --- | --- | --- | --- | --- | --- |
|  | **HR (95% CI)** | **P** | **HR (95% CI)** | **P** | **HR (95% CI)** | **P** |
| **All-cause mortality** | | | | | | |
| Q1 | Reference | | Reference | | Reference | |
| Q2 | 1.47(1.28-1.70) | **<.001** | 1.16(1.01-1.34) | **0.039** | 1.12(0.97-1.30) | 0.109 |
| Q3 | 1.64(1.42-1.88) | **<.001** | 1.23(1.07-1.41) | **0.004** | 1.18(1.02-1.35) | **0.026** |
| Q4 | 1.88(1.64-2.15) | **<.001** | 1.42(1.24-1.63) | **<.001** | 1.28(1.11-1.48) | **0.001** |
| **Cardiovascular mortality** | | | | | | |
| Q1 | Reference | | Reference | | Reference | |
| Q2 | 1.54(1.17-2.03) | **0.002** | 1.21(0.92-1.60) | 0.181 | 1.17(0.88-1.55) | 0.271 |
| Q3 | 1.72(1.31-2.26) | **<.001** | 1.28(0.97-1.68) | 0.084 | 1.25(0.95-1.65) | 0.119 |
| Q4 | 2.32(1.79-3.02) | **<.001** | 1.74(1.34-2.27) | **<.001** | 1.57(1.20-2.06) | **0.001** |

Abbreviation: TyG: triglyceride-glucose; FLI: fatty liver index; WC: waist circumference; HR: hazard ratio; CI: confidence interval; Q: quartile.

Model 1: unadjusted; Model 2: adjusted for age, gender, race; Model 3: adjusted for age, gender, race, marital status, educational level, energy intakes, poverty income ratio, smoking status, alcohol use, CVD, CKD, cancer, AST, ALT, and TC.

**Table S9**. Sensitive analysis to evaluate the association between TyG-WHtR index with the mortality outcomes of adults with FLI ≥ 30.

| **Subgroup** | **Model 1** | | **Model 2** | | **Model 3** | |
| --- | --- | --- | --- | --- | --- | --- |
|  | **HR (95% CI)** | **P** | **HR (95% CI)** | **P** | **HR (95% CI)** | **P** |
| **All-cause mortality** | | | | | | |
| Q1 | Reference | | Reference | | Reference | |
| Q2 | 1.39(1.20-1.60) | **<.001** | 1.16(1.00-1.34) | 0.054 | 1.11(0.96-1.29) | 0.159 |
| Q3 | 1.80(1.57-2.07) | **<.001** | 1.43(1.24-1.64) | **<.001** | 1.30(1.12-1.50) | **<.001** |
| Q4 | 2.02(1.76-2.32) | **<.001** | 1.65(1.43-1.91) | **<.001** | 1.45(1.26-1.68) | **<.001** |
| **Cardiovascular mortality** | | | | | | |
| Q1 | Reference | | Reference | | Reference | |
| Q2 | 1.35(1.01-1.80) | **0.043** | 1.13(0.85-1.52) | 0.404 | 1.10(0.82-1.47) | 0.533 |
| Q3 | 1.99(1.52-2.61) | **<.001** | 1.59(1.21-2.10) | **0.001** | 1.45(1.10-1.92) | **0.009** |
| Q4 | 2.38(1.83-3.10) | **<.001** | 2.00(1.52-2.62) | **<.001** | 1.74(1.32-2.30) | **<.001** |

Abbreviation: TyG: triglyceride-glucose; FLI: fatty liver index; WHtR: waist to height ratio; HR: hazard ratio; CI: confidence interval; Q: quartile.

Model 1: unadjusted; Model 2: adjusted for age, gender, race; Model 3: adjusted for age, gender, race, marital status, educational level, energy intakes, poverty income ratio, smoking status, alcohol use, CVD, CKD, cancer, AST, ALT, and TC.
